# Acute lymphocytic crisis following herpes simplex type 1 virus hepatitis in a nonimmunocompromised man: a case report

**DOI:** 10.4076/1752-1947-3-7492

**Published:** 2009-08-03

**Authors:** Sotiris Plastiras, Ourania Kampessi

**Affiliations:** 1 Department of Pathophysiology, University of Athens School of Medicine, Laiko University Hospital, 11527 Athens, Greece

## Abstract

**Introduction:**

An increase in circulating lymphocytes can be seen following infections such as infectious mononucleosis and pertussis, or in lymphoproliferative disorders such as acute and chronic lymphocytic leukemia. Acute lymphocytic crisis following herpes simplex virus hepatitis has not been described in the literature.

**Case presentation:**

A 52-year-old man was admitted to our hospital reporting low-grade fever for the previous seven days, and fatigue. During the fifth day of hospitalization, the patient developed a lymphocytic crisis and, after further tests the patient was diagnosed as having herpes simplex virus hepatitis.

**Conclusion:**

This case report shows that herpes simplex virus type 1 is a possible cause of an acute lymphocytic crisis similar to other well known infectious agents such as Epstein–Barr virus, cytomegalovirus, human immunodeficiency virus, human herpes virus type 6, adenovirus, toxoplasma and human T-cell lymphotropic virus. Furthermore, this case report expands the clinical spectrum of herpes simplex virus hepatitis, since it is reported in a nonimmunocompromised patient presenting with atypical acute lymphocytic syndrome.

## Introduction

An increase in circulating lymphocytes can be seen following infections such as infectious mononucleosis and pertussis [1], or in lymphoproliferative disorders such as acute and chronic lymphocytic leukemia. We report on a patient whose laboratory findings indicated an acute lymphocytic crisis after herpes simplex virus (HSV) hepatitis.

## Case presentation

A 52-year-old, Caucasian man of Greek origin was admitted to our hospital reporting low-grade fever for the previous 7 days, and fatigue. His past medical history was unremarkable. Clinical examination revealed a low-grade hepatomegaly (1 to 2cm) and two palpable lymph nodes at the left lateral neck with a soft constitution, movable and mildly sensitive when palpated.

The results of the laboratory tests carried out on admission are shown in Table 1. Direct and indirect antiglobulin tests were negative; C-reactive protein (CRP) and erythrocyte sedimentation rate (ESR) were 37.2mg/L and 30mm/hour, respectively. Immunologic tests and urine analysis were normal. Blood and urine cultures and the Mantoux test were negative. Serologic findings including brucella, rickettsia and coxsiella burnetii, borrelia burgdorferi, Epstein–Barr virus (EBV), cytomegalovirus (CMV), herpes virus zoster, toxoplasma, coxsackie, mycoplasma pneumoniae, hepatitis B virus, hepatitis A virus, adenovirus, echo virus, human immunodeficiency virus (HIV), and herpes virus type 6 using ELISA were all normal. Polymerase chain reaction (PCR) for EBV and antistreptolysin titer was negative. Positive results were HSV type 1 IgG; 5.9 (Positive > 1.1); HSV type 2 IgG; 0.1 (Positive > 1.1) and HSV type 1 IgM; 4.6 (Positive > 1.1); HSV type 2; 0.5 (Positive > 1.1).

**Table 1:** Serial laboratory findings over 2 months

	Hct	Hb	WBC	RBC	PLT	Urea	Creat.	tBil	dBil	ALP	AST	ALT	LDH	CPK	γGT
On admission	45.2	15.7	4.8 × 10^3^ (53.7/40.7)	5.8 × 10^6^	147 × 10^3^	24	1.1	2.78	2.34	444	126	275	864	40	245
Fifth day	43	14.8	12.1 × 10^3^ (32.8/57.8)	5.6 × 10^6^	180 × 10^3^	26	1.0	3.0	2.6	600	162	334	880	46	280
Seventh day	43	14.8	25 × 10^3^ (11.6/78.9)	5.6 × 10^6^	200 × 10^3^	32	0.9	3.4	2.8	1048	189	496	900	42	351
Discharge	42.4	14.3	17.4 × 10^3^ (12/78)	5.4 × 10^6^	213 × 10^3^	30	0.9	1.13	0.8	932	176	423	829	49	324
One week	44.5	14.7	11 × 10^3^ (14/78)	5.4 × 10^6^	213 × 10^3^	28	1.0	0.9	0.3	298	84	227	716	46	287
One month	44.1	14.6	6.1 × 10^3^	5.5 × 10^6^	276 × 10^3^	30	1.0	0.6	0.3	73	24	32	295	49	46
Two months	44	14.8	6.8 × 10^3^ (60/25)	5.2 × 10^6^	250 × 10^3^	28	0.8	0.6	0.2	70	22	22	290	42	35

Abbreviations: Hct, hematocrit (%); Hb, hemoglobin (g/dL); WBC, white blood cells (K/μL); N, neutrophils (%); L, lymphocytes (%); RBC, red blood cells (M/μL); PLT, platelet count (K/μL); creat, creatinine (mg/dL); tBil, total bilirubin (mg/dL); dBil, direct bilirubin (mg/dL); ALP, alkaline phosphatase (U/L); AST, aspartate aminotransferase (U/L); ALT, alanine aminotransferase (U/L); LDH, lactate dehydrogenase (U/L); CPK, creatine phosphokinase (U/L); γGT, gamma-glutamyl transferase (U/L).

Electrocardiogram (ECG) and chest X-ray were normal. Abdominal ultrasonography revealed low-grade hepatomegaly. Neck ultrasonography showed two lymph nodes on the left side of the neck to be 32mm and 26mm in diameter, with a central hematosis of inflammatory etiology.

During the first four days the patient was in good condition with low-grade fever in the afternoons and the laboratory values indicated a fall in inflammation indexes. During the fifth day of hospitalization, acute leukocytosis along with a remarkable lymphocytosis was noted. The peripheral blood smear revealed the presence of atypical activated lymphocytes, containing significantly elevated cytotoxic/suppressor (CD8) T cells and helper (CD4) T cells. Simultaneously, we noticed a daily rise of liver enzymes and a gradual decrease in bilirubin values. Despite the laboratory findings of cholestatic hepatitis and acute lymphocytic crisis no change in his clinical condition occurred.

To exclude the possibility of a lymphoproliferative syndrome we carried out further diagnostic tests. A liver biopsy revealed widening of some of the portal spaces with moderate inflammatory infiltration mainly from lymphocytes partially altered with diffuse parenchyma. Some of the lobar intrahepatic ducts presented with mild degenerative changes and some inflammation. The parenchyma revealed inflammatory infiltration of sinusoids from lymphocytes and slight proliferation of the Kupffer cells with some focal necrosis and apoptosis. Coagulative necrosis surrounded by hepatocytes, with typical viral inclusions of HSV were also noted. There was also a small number of lymphohistiocytic and rare epithelial-like granulomas plus an increase in the mitotic activity of hepatic cells and Kupffer cells, changes suggestive of viral hepatitis. Bone marrow aspiration, which was performed on the sixth day of hospitalization, showed poor maturation of red blood cells and increased lymphocytes. Bone marrow biopsy revealed the presence of multiple granulomatic bodies, which were surrounded by a moderate number of small T-reactive lymphocytes. The number of B-lymphocytes was significantly decreased and flow cytometry of the marrow indicated a decrease of the CD4 level and a rise of the CD8 level. Also, the monoclonal test of lymphocytes after a molecular study of a bone sample did not trace rearrangement of the T-cell receptor. Abdominal computed tomography (CT) scanning showed a low-grade hepatomegaly. DNA examination of the bone sample using PCR for HSV, Epstein–Barr virus and cytomegalovirus were negative. We did not perform PCR for the viruses above from the liver sample because of the inadequate quantity that was aspirated. Considering all these mentioned above, while the possibility of a lymphoproliferative syndrome was excluded, we concluded that the patient had HSV hepatitis, from which an acute lymphocytic syndrome followed.

After 15 days of hospitalization the patient was in good condition without any medication. He had no fever and he reported a sense of weakness at the bottom of his limbs as in chronic fatigue syndrome after a viral infection. Neurological examination results were normal. From this point gradual improvement in the laboratory findings was observed. One week after discharge, he reported a mild sense of weakness and the laboratory findings were as shown in Table 1. One month after the first follow-up visit, his weakness had totally gone and the laboratory findings are shown in Table 1. CRP was 49 mg/L Two months later, the patient is free of symptoms and is fully active. HSV type 1 IgG was 3.5 (positive >1.1); HSV type 1 IgM was 0.4 (positive >1.1).

## Discussion

Hepatitis is an unusual manifestation of HSV infection. HSV hepatitis is a difficult diagnosis to establish, and the infection is often fatal. Kaufman et al. [2] described one case of HSV hepatitis and reviewed 51 cases in the literature. These authors concluded that impaired immunity resulting from pregnancy, malignancy, immunosuppression or inhalational anesthetic use may be predisposing factors [2].

In the absence of a common cause of liver failure, histology and immunostaining of transjugular liver biopsy specimens, can establish or confirm the diagnosis of herpetic hepatitis. HSV hepatitis has been described during both recurrent and primary HSV infection in immunocompromised hosts [3,4]. In immunocompetent hosts, however, only primary infections have been associated with hepatitis [5]-[7]. In most cases described by Kaufman et al. [2], a positive diagnosis of HSV hepatitis was made too late. However, some features should be emphasized: 1) fever was present in all cases; 2) herpetic lesions were absent; 3) leukopenia was present in only two out of five patients; 4) aminotransferase levels were increased in all but one case; and 5) virologic and histologic results were concordant, but were available too late. As far as we can conclude from appropriate and extensive searching, our patient was not immunocompromised and HSV hepatitis presented with the unusual laboratory finding of acute lymphocytic crisis.

Acute lymphocytic syndrome is generally considered as a common condition due to various causes. Lymphocytosis can be either reactive or malignant [8]. Reactive lymphocytosis refers to lymphocytosis in patients without a history of a hematologic disorder, who have a medical condition likely to be associated with lymphocytosis, and in whom the lymphocyte count normalizes, or is expected to normalize, within two months of resolution of this condition. Examples of causes include viral infection and pertussis. Malignant lymphocytosis refers to lymphocytosis and an established diagnosis of an acute or chronic lymphoproliferative disorder. Examples are chronic lymphocytic leukemia (CLL), acute lymphoblastic leukemia (ALL), and large granular lymphocyte (LGL) leukemia.

Reactive lymphocytosis can be confused with malignant lymphocytosis, for example, the various leukemic states, by an inexperienced observer viewing the Wright-Giemsa stained blood smear. In general, reactive lymphocytosis is due to the presence in the peripheral blood of one of two types of lymphocytes: an absolute increase in mature normal-appearing small lymphocytes, as seen in pertussis and infectious lymphocytosis, and an absolute increase in larger lymphocytes with abundant basophilic cytoplasm and a large irregularly shaped nucleus containing a rare nucleolus. Infectious mononucleosis during the second and third week of illness results in a marked increase in these larger forms of lymphocytes (so-called atypical, transformed or lymph-variant lymphocytes). A number of different types of lymphoid cells may appear in the peripheral blood in the various malignant lymphoproliferative disorders. The early phases of B-cell, T-cell or NK-cell lymphoproliferative malignancies in adults can mimic these benign forms of polyclonal or reactive lymphocytosis and that was a clue in our patient. Bone marrow, immunophenotyping, gene rearrangement studies, and/or cytogenetic studies are required to differentiate chronic lymphocytic leukemia or, sometimes, T-cell leukemia, from benign lymphocytosis [9]. The usual cause of reactive lymphocytosis is one of a variety of viral infections most frequently observed in children and young adults. EBV is the major cause of infectious mononucleosis [10] which is associated with a marked reactive "atypical" lymphocytosis. These atypical lymphocytes peak during the second and third week of illness, and persist for as long as two months. Although EBV infects B-lymphocytes, the reactive lymphocytosis in the blood is due to absolute increases in T-lymphocytes, predominantly of the CD8 subtype [11].

A clinical picture resembling infectious mononucleosis (mononucleosis syndrome) may be caused by a number of infectious agents other than EBV. The two most important entities to include in the differential diagnosis of the mononucleosis syndrome are infections with CMV [12] and the human immunodeficiency virus (HIV-1). Other infections have been implicated as occasional etiologies of the mononucleosis syndrome, including human herpes virus type 6, adenovirus type 12 and toxoplasmosis. Other viral illnesses such as human T-cell lymphotropic virus (HTLV-1) infection have resulted in a self-limited immature T-cell lymphocytosis as high as 20,000/µl, evolving into a mature T-cell lymphocytosis with a CD4/CD8 ratio of 4.5:1 [13]. Patients with mumps, varicella, influenza, hepatitis, rubella or measles infections usually have reactive lymphocytosis as a hematologic feature of their illness [14].

In our case, the qualitative immunoenzymatic determination of IgG-class and IgM-class antibodies against HSV type 1 and type 2 was based on the ELISA (enzyme-linked immunosorbent assay) technique. The diagnostic specificity for both IgG-class and IgM-class was >98% and the diagnostic sensitivity was 95.3%. Cross-reactivity with adenovirus, CMV, EBV, echinococcus, HBV, influenza A-B, mycoplasma pneumoniae, picorna, syphilis, rubella, toxoplasma and varicella zoster virus was negative (these technical characteristics refer to the kit that is used in our hospital (Novatec (Dietzenbach, Germany) HSV type 1 and type 2 IgG, IgM-ELISA) [15].

## Conclusion

This case report shows that HSV type 1 is a possible cause of acute lymphocytic crisis similar to other well known infectious agents such as EBV, CMV, HIV, human herpes virus type 6, adenovirus, toxaplasma and HTLV-1. This case report expands the clinical spectrum of HSV hepatitis, since it is reported in a non-immunocompromised patient presenting with atypical acute lymphocytic syndrome.

## Abbreviations

ALL: acute lymphoblastic leukemia; CLL: chronic lymphocytic leukemia; CMV: cytomegalovirus; CRP: C-reactive protein; CT: computed tomography; EBV: Epstein–Barr virus; ECG: electrocardiogram; ESR: erythrocyte sedimentation rate; HIV: human immunodeficiency virus; HSV: herpes simplex virus; LGL: large granular lymphocyte leukemia; PCR: polymerase chain reaction.

## Consent

Written informed consent was obtained from the patient for publication of this case report and accompanying images. A copy of the written consent is available for review by the Editor-in-Chief of this journal.

## Competing interests

The authors declare that they have no competing interests.

## Authors' contributions

The study was designed and the case report written by both SP and OK. OK was in charge of patient care and SP the histologic examination.
